# Trends in and factors associated with the adoption of digital aids for smoking cessation and alcohol reduction: A population survey in England

**DOI:** 10.1016/j.drugalcdep.2019.107653

**Published:** 2019-12-01

**Authors:** Olga Perski, Sarah E. Jackson, Claire Garnett, Robert West, Jamie Brown

**Affiliations:** Department of Behavioral Science and Health, University College London, 1-19 Torrington Place, London, WC1E 6BT, UK

**Keywords:** Smoking, Excessive alcohol consumption, Quit attempts, Digital aids, Diffusion of innovation

## Abstract

**Background:**

Digital smoking cessation and alcohol reduction aids are widely available in England. To estimate their public health impact, researchers need to consider their adoption in the target population. We assessed adoption rates, and characteristics of adopters, of digital smoking cessation and alcohol reduction aids in England.

**Methods:**

3655 smokers and 2998 high-risk drinkers (defined as a score of >4 on the Alcohol Use Disorders Identification Test-Consumption; AUDIT-C) who had made a past-year quit/reduction attempt were surveyed as part of the Smoking and Alcohol Toolkit Studies between January 2015-October 2018. Respondents provided information on socio-demographic characteristics and whether they had used a digital aid in a recent quit/reduction attempt.

**Results:**

2.7 % (95 % CI 2.2%–3.0%) of smokers and 3.6 % (95 % CI 2.9%–4.0%) of drinkers who had made a past-year quit/reduction attempt (26.9 % and 15.3 %, respectively) had used a digital aid. Survey year was not significantly associated with use in smokers or drinkers. None of the baseline characteristics were significantly associated with the use of a digital aid in smokers. Drinkers with high motivation to reduce alcohol consumption (OR_adj_ = 2.49, 95 % CI 1.63–3.77, *p* <  .001) and higher AUDIT scores (OR_adj_ = 1.07, 95 % CI 1.03–1.11, *p* < .001) had greater odds of adoption.

**Conclusions:**

Digital smoking cessation and alcohol reduction aids are rarely used by smokers or high-risk drinkers attempting to quit/cut down in England, indicating that most of the target population is not being reached. Despite overall digital access improving, adoption rates remained similarly low between 2015–2018.

## Highlights

• Less than 5 % of smokers and drinkers in England who have made a quit/reduction attempt used a digital cessation aid.
• Adoption rates remained similarly low between 2015 and 2018.
• Drinkers with high motivation to reduce consumption had greater odds of adoption.
• Drinkers with greater consumption and related problems had greater odds of adoption.

## 1 Introduction

Digital aids (also referred to in the literature as ‘digital interventions’ or ‘digital health technologies’) can be defined as “a product or service that uses computer technology to promote behaviour change” (West and Michie, 2016). These aids can include websites and applications (‘apps’) accessed via smartphones, tablets, personal digital assistants (PDAs) or wearable devices. Evidence from randomised controlled trials (RCTs) indicate that digital aids can successfully help people to quit smoking and reduce excessive alcohol consumption; however, effect sizes are typically small-to-medium (Kaner et al., 2017; Taylor et al., 2017). To estimate the potential public health impact of digital aids for smoking cessation and alcohol reduction, researchers also need to consider their reach within the target population. A highly effective intervention that only reaches a small proportion of the target population is likely to have a smaller public health impact than an intervention with a small effect that reaches a large proportion of the target population (Glasgow et al., 1999). Although effect sizes tend to be small, digital aids have the potential to reach a large proportion of the target population as internet access and personal ownership of digital devices are now widespread (Office for National Statistics, 2018; Perrin and Duggan, 2015). Moreover, digital aids can help overcome geographical barriers to and stigma associated with help-seeking in person (Ritterband and Tate, 2009).

We currently know little about the reach of digital aids for smoking cessation and alcohol reduction in the general population of adult smokers and high-risk drinkers in England or globally. In a random probability sample of smokers in Canada in 2006–2007, 40 % of respondents said that they were interested in using a digital aid for smoking cessation (Cunningham, 2008). Estimates from 2012 indicated that just under 50 % of smokers in England were interested in using a digital aid for smoking cessation, but that less than 0.5 % had used such support in a recent quit attempt (Brown et al., 2013). In the American National Cancer Institute’s Health Information National Trends Survey (HINTS), 19.8 % of current smokers and 15.9 % of excessive drinkers reported that they had used the internet to access some form of behavioural support (not necessarily specific to smoking or drinking) in the past year (Shahab et al., 2014). A study from 2016 examined the use of aids for smoking cessation and alcohol reduction (e.g. pharmacological, face-to-face, digital) in England, but only reported results stratified by sociodemographic characteristics and did not focus on the adoption of digital aids (Beard et al., 2016). In this paper, we seek to provide up-to-date estimates of the rate of adoption, and characteristics of adopters, of digital aids for smoking cessation and alcohol reduction in a representative sample of smokers and high-risk drinkers in England. We focus solely on high-risk (as opposed to regular) drinkers as this group is more directly comparable with current smokers and the greater priority for public health research.

According to the ‘Diffusion of Innovations’ theory, diffusion is a process by which new ideas or innovations spread over time among members of a social network via mass media or interpersonal communication (Rogers, 1995). Although the diffusion process often unfolds over a lengthy time period, innovations with a clear relative advantage compared with existing products (i.e. where rewards to individuals are immediate) tend to have a faster rate of adoption than preventative innovations where rewards are reaped at some point in the future (Rogers, 2002). As it typically takes several months for users to achieve the intended outcomes of digital aids for smoking cessation and alcohol reduction, such innovations might diffuse relatively slowly. To date, we know little about the rate of adoption of digital aids within the population of smokers and drinkers in England, whether this has changed over time and whether adoption rates differ between smokers and drinkers.

Importantly, the process of diffusion often occurs unequally across different social groups: it is often older, less educated and more disadvantaged groups who are the slowest to adopt innovations (Office for National Statistics, 2018; Perrin and Duggan, 2015). Internet access and personal smartphone ownership has grown rapidly in the past decade, with 84–89% of adults in the United Kingdom (UK) and the United States (US) having access to the internet, and 64–68% owning a smartphone in 2015–2016 (Office for National Statistics, 2016; Perrin and Duggan, 2015); however, the rate of adoption has been slower in older adults and in disadvantaged groups. Similarly, data from the US HINTS suggest that early adopters of health and fitness apps tend to be younger, more affluent and more highly educated than non-adopters (Carroll et al., 2017). In an international sample of drinkers recruited via the Global Drug Survey, digital aids were the preferred source of support for respondents from Australia, New Zealand and the UK, lower-risk drinkers and those without a mental health condition (Davies et al., 2019). We currently lack knowledge as to whether particular sociodemographic or smoking/drinking characteristics are associated with the adoption of digital aids for smoking cessation and alcohol reduction in England. If so, this information could be used to inform targeted strategies to accelerate the diffusion process. Therefore, we aimed to address the following research questions (RQs):

1 What proportion of smokers and high-risk drinkers who have made at least one past-year quit/reduction attempt report having used a digital aid (i.e. a website or an app on a smartphone, tablet or PDA)?
2 Does the proportion of individuals who report having used a digital aid in a recent quit/reduction attempt differ between smokers and drinkers?
3 Has the proportion of smokers or drinkers who report having used a digital aid in a recent quit/reduction attempt changed between 2015 and 2018?
4 Among smokers, is survey year, age, sex, social grade, frequency of internet access, cigarettes per day or motivation to stop independently associated with reports of having used a digital aid in a recent quit attempt?
5 Among high-risk drinkers, is survey year, age, sex, social grade, frequency of internet access, patterns of alcohol consumption or motivation to reduce alcohol consumption independently associated with reports of having used a digital aid in a recent reduction attempt?

## 2 Methods

### 2.1 Study design and setting

The study protocol and analysis plan were pre-registered on the Open Science Framework (osf.io/ztgw6). The STROBE guidelines were used in the design and reporting of this study (Von Elm et al., 2007). The data were collected as part of the ongoing Smoking and Alcohol Toolkit Studies (STS and ATS), which involve monthly, face-to-face, computer-assisted household surveys of adults aged 16+ in England (Beard et al., 2015; Fidler et al., 2011). The sample is a hybrid of a random probability and quota sample, which results in a sample that is representative of the adult population of smokers and drinkers in England. Interviewers travel to selected output areas and perform computer-assisted interviews with one household member aged 16+ years until quotas based on factors influencing the probability of being at home (i.e. working status, age and gender) are fulfilled. In this hybrid form of random probability and quota sampling (which is considered superior to conventional quota sampling), the choice of households to approach is limited by the random allocation of small output areas. Rather than being sent to specific households in advance, interviewers can choose which households within these areas are most likely to fulfil their quotas. Unlike random probability sampling, where interviewers have no choice as to the households sampled and can record responses at each address, it is not appropriate to record response rates in the STS and ATS. Informed consent is obtained prior to each interview. Ethical approval was granted by UCL’s Research Ethics Committee (0498/001).

### 2.2 Study population

Data included in the present study were collected from respondents surveyed between January 2015 (the first full year when the question about use of digital aids was included in both the STS and the ATS) and October 2018 (the latest wave of data available). Respondents were aged 16+ years at the time of the survey and were included in the analyses if they: i) currently smoked cigarettes or any other tobacco product daily or occasionally or were classified as a ‘high-risk’ drinker (defined by a score of >4 on the 3-item Alcohol Use Disorders Identification Test-Consumption; AUDIT-C (Babor et al., 2001)) at the time of the survey; and ii) reported having made at least one serious attempt to quit smoking or reduce their alcohol consumption in the past year. This deviated from the pre-registered study protocol, in which we had specified that recent ex-smokers would also be included. Respondents who have quit smoking successfully are not asked about motivation to stop in the STS and could therefore not be included in the main analyses. Instead, sensitivity analyses were carried out (detailed in section 2.4).

### 2.3 Measures

In smokers, the outcome variable was the proportion of smokers who reported the use of a digital aid in a recent quit attempt. This was measured by asking: “Which, if any, of the following did you try to help you stop smoking during the most recent serious quit attempt?” Responses were coded 1 for those who selected one of the following options and 0 otherwise: “Visited www.nhs.uk/smokefree website”, “Visited a website other than Smokefree” or “Used an application on a handheld computer (smartphone, tablet or PDA)”.

The predictor variables were: survey year (2015, 2016, 2017, 2018); age (16–24 years, 25–34 years, 35–44 years, 45–54 years, 55–64 years, 65+ years); sex (male, female); social grade, assessed by the British National Readership Survey’s Social Grade Classification Tool (IPSOS MediaCT, 2009), with responses dichotomised into low (C2DE) and high (ABC1) social grade. These groupings are frequently interpreted by researchers to represent the working and middle classes, respectively. This occupational measure of social grade is a valid indicator of socioeconomic status that is widely used in research in England. It has been identified as particularly relevant in the context of tobacco smoking (Kotz and West, 2009) and alcohol consumption (Beard et al., 2019); frequency of internet access; cigarettes per day (converted to daily consumption for non-daily smokers who reported the number of cigarettes smoked per week); and motivation to stop smoking. Frequency of internet access was measured by asking: “How frequently do you access the internet?”. The response options were: 1) Never but I do not have access; 2) Never but I have access; 3) Less than around once a month; 4) Around once a month; 5) 2 or 3 times a month; 6) Around once a week; 7) 2 or 3 times a week; 8) 4 or 5 times a week; 9) Around once a day; 10) Several times a day. For ease of interpretation, response options were collapsed into Never (1–2), Rarely (3–8) and Daily (9–10). Motivation to stop smoking was measured by the Motivation to Stop Scale (MTSS) (Kotz et al., 2013), which asks: “Which of the following best describes you?” The response options were: 1) “I don’t want to stop smoking”; 2) “I think I should stop smoking but don’t really want to”; 3) “I want to stop smoking but haven’t thought about when”; 4) “I REALLY want to stop smoking but I don’t know when I will”; 5) “I want to stop smoking and hope to soon”; 6) “I REALLY want to stop smoking and intend to in the next 3 months”; 7) “I REALLY want to stop smoking and intend to in the next month”. To aid interpretation, responses were dichotomised into low (1–5) and high (6–7) motivation, as is often done in studies using this variable (Hitchman et al., 2015; Jackson et al., 2018).

In drinkers, the outcome variable was the proportion of high-risk drinkers who reported the use of a digital aid in a recent attempt to reduce their alcohol consumption. This was measured by asking: “Which, if any, of the following did you use to try to help restrict your alcohol consumption during the most recent attempt?” Responses were coded 1 for those who selected one of the following options and 0 otherwise: “Visited a website for help with drinking” or “Used an application (‘app’) on a handheld computer (smartphone, tablet, PDA)”.

The predictor variables were: survey year; age; sex; social grade; frequency of internet access; alcohol consumption; and motivation to reduce drinking. Alcohol consumption was measured by the Alcohol Use Disorders Identification Test (AUDIT), a 10-item measurement of alcohol consumption, drinking behaviour and alcohol-related problems that provides a score ranging from 0 to 40 (Babor et al., 2001). Motivation to reduce drinking was measured by the MTSS, adapted for use in drinkers. The response options were: 1) “I don’t want to cut down on drinking alcohol”; 2) “I think I should cut down on drinking alcohol but don’t really want to”; 3) “I want to cut down on drinking alcohol but haven’t thought about when”; 4) “I REALLY want to cut down on drinking alcohol but I don’t know when I will”; 5) “I want to cut down on drinking alcohol and hope to soon”; 6) “I REALLY want to cut down on drinking alcohol and intend to in the next 3 months”; 7) “I REALLY want to cut down on drinking alcohol and intend to in the next month”. To aid interpretation, responses were dichotomised into low (1–5) and high (6–7) motivation.

### 2.4 Data analysis

Analyses were conducted in R v.3.5.1 using the *survey* package. Data included in the analyses concerned with prevalence (i.e. RQs 1–3) were weighted using the rim (marginal) technique (Sharot, 1986) to match the sample to the proportions of the English population profile on the dimensions of age, social grade, region, tenure, ethnicity and working status within sex. The dimensions are derived monthly from a combination of the English 2011 census Office for National Statistics mid-year estimates, and an annual random probability survey conducted for the National Readership Survey. Participants with missing data for any of the variables in the analyses were excluded.

The proportions of smokers and high-risk drinkers who reported that they had used a digital aid in a recent quit/reduction attempt and 95 % confidence intervals (CIs) were estimated using the likelihood method, which uses the Rao-Scott scaled χ^2^ distribution for the log-likelihood from a binormal distribution. This deviated from the pre-registered analysis plan, which specified the use of the Wilson Score Interval, but this estimation method was not available in the *survey* package. The proportions of smokers and drinkers who reported that they had used a digital aid in a recent quit/reduction attempt were compared using a two-proportion *z*-test. A sensitivity analysis was conducted to estimate the proportion of recent ex-smokers (i.e. respondents who had successfully quit within the past 12 months) who reported that they had used a digital aid in a recent quit attempt.

The associations between survey year and the use of a digital aid in a recent quit/reduction attempt were assessed in univariable logistic regression analyses. A sensitivity analysis was conducted to examine the associations between survey year and the use of a digital aid in a recent quit attempt among recent ex-smokers.

In smokers, the associations between survey year, age, sex, social grade, frequency of internet access, motivation to stop, cigarettes per day and having used a digital aid in a recent quit attempt were assessed in a multivariable logistic regression analysis. In drinkers, the associations between survey year, age, sex, social grade, frequency of internet access, AUDIT score, motivation to reduce alcohol consumption and having used a digital aid in a recent attempt to reduce drinking were assessed in a multivariable logistic regression analysis.

## 3 Results

Of 13,985 smokers surveyed between January 2015 and October 2018, 3761 (26.9 %) had made at least one quit attempt. Data on motivation to stop and cigarettes per day were missing for 106 respondents, yielding a total sample size of 3655 (97.2 %) respondents with complete data on all other variables of interest. Of a total of 19,995 high-risk drinkers surveyed between the same time period, 3061 (15.3 %) had made at least one recent attempt to reduce their drinking. Data on motivation to reduce drinking and the AUDIT were missing for 63 respondents, yielding a total sample size of 2998 (97.9 %) respondents with complete data on all other variables of interest. Smokers’ and drinkers’ characteristics are reported in Table 1, Table 2, respectively.

**Table 1:** Smokers’ characteristics in the unweighted and weighted datasets, Odds Ratios (ORs) from the weighted univariable analysis, and adjusted Odds Ratios (OR_adj_) from the unweighted multivariable analysis.

	Smokers^a^ (*N* = 3655)	Smokers^b^ (*N* = 3765)	% Used a digital aid in recent attempt^b^ (*n/N*)	OR (95% CI)	OR_adj_ (95% CI)
**Used a digital aid in recent attempt, *n* (%)**					
No	3558 (97.3%)	3662 (97.3%)	–	–	–
Yes	97 (2.7%)	103 (2.7%)	–	–	–

**Survey year, *n* (%)**					
2015	1049 (28.7%)	1041 (27.6%)	3.1% (32/1041)	1.00	1.00
2016	901 (24.6%)	949 (25.2%)	3.1% (29/949)	0.99 (0.56-1.75)	0.98 (0.57-1.66)
2017	958 (26.2%)	1005 (26.7%)	2.7% (27/1005)	0.86 (0.48-1.52)	0.90 (0.53-1.54)
2018	747 (20.4%)	770 (20.5%)	1.7% (13/770)	0.56 (0.28-1.13)	0.63 (0.32-1.19)

**Age, *n* (%)**					
16-24	705 (19.3%)	697 (18.5%)	2.7% (19/697)	–	1.00
25-34	849 (23.2%)	975 (25.9%)	3.2% (31/975)	–	1.10 (0.61-1.99)
35-44	655 (17.9%)	742 (19.7%)	3.6% (27/742)	–	1.31 (0.72-2.41)
45-54	617 (16.9%)	657 (17.5%)	2.3% (15/657)	–	0.82 (0.39-1.64)
55-64	466 (12.7%)	403 (10.7%)	1.7% (7/403)	–	0.69 (0.28-1.55)
65+	363 (9.9%)	291 (7.7%)	1.0% (3/291)	–	0.62 (0.19-1.62)

**Sex, *n* (%)**					
Men	1818 (49.7%)	1887 (50.1%)	3.0% (57/1887)	–	1.00
Women	1837 (50.3%)	1878 (49.9%)	2.4% (45/1878)	–	0.76 (0.50-1.15)

**Social grade, *n* (%)**					
C2DE	2098 (57.4%)	2231 (59.3%)	2.2% (50/2231)	–	1.00
ABC1	1557 (42.6%)	1534 (40.7%)	3.4% (52/1534)	–	1.47 (0.97-2.24)

**Frequency of internet access, *n* (%)**					
Never	354 (9.7%)	301 (8.0%)	1.0% (3/301)	–	1.00
Rarely	279 (7.6%)	269 (7.1%)	1.1% (3/269)	–	0.81 (0.16-3.74)
Frequently	3022 (82.7%)	3194 (84.8%)	3.0% (96/3194)	–	1.85 (0.70-6.40)

**Motivation to stop, *n* (%)**					
Low	2477 (67.8%)	2561 (68.0%)	2.9% (74/2561)	–	1.00
High	1178 (32.2%)	1203 (32.0%)	2.3% (28/1203)	–	0.82 (0.52-1.27)
**Cigarettes per day, mean (*SD*)**	10.5 (*7.9*)	10.4 (*8.3*)	–	–	1.00 (0.97-1.03)

*Note.*^a^ Unweighted; ^b^ Weighted using the rim (marginal) technique to match the sample to the proportions of the English population profile on the dimensions of age, social grade, region, tenure, ethnicity and working status within sex; * *p* < 0.05; ** *p* < 0.01; *** *p* < 0.001.

**Table 2:** Drinkers’ characteristics in the unweighted and weighted datasets, Odds Ratios (ORs) from the weighted univariable analysis, and adjusted Odds Ratios (OR_adj_) from the unweighted multivariable analysis.

	High-risk drinkers^a^ (*N* = 2998)	High-risk drinkers^b^ (*N* = 3198)	% Used a digital aid in recent attempt^b^ (*n/N*)	OR (95% CI)	OR_adj_
**Used a digital aid in recent attempt, *n* (%)**					
No	2895 (96.6%)	3084 (96.4%)	–	–	–
Yes	103 (3.4%)	114 (3.6%)	–	–	–

**Survey year, *n* (%)**					
2015	724 (24.1%)	839 (26.2%)	3.1% (26/839)	1.00	1.00
2016	765 (25.5%)	806 (25.2%)	4.5% (36/806)	1.47 (0.81-2.67)	1.43 (0.82-2.55)
2017	884 (29.5%)	905 (28.3%)	4.3% (39/905)	1.41 (0.79-2.52)	1.51 (0.88-2.65)
2018	625 (20.8%)	649 (20.3%)	2.0% (13/649)	0.67 (0.32-1.41)	0.70 (0.35-1.38)

**Age, *n* (%)**					
16-24	436 (14.5%)	404 (12.6%)	2.7% (11/404)	–	1.00
25-34	430 (14.3%)	535 (16.7%)	5.2% (28/535)	–	1.96 (0.97-4.17)
35-44	502 (16.7%)	634 (19.8%)	3.2% (20/634)	–	1.31 (0.62-2.86)
45-54	628 (20.9%)	756 (23.6%)	4.4% (33/756)	–	1.62 (0.81-3.43)
55-64	589 (19.6%)	540 (16.9%)	3.9% (21/540)	–	1.60 (0.77-3.45)
65+	413 (13.8%)	329 (10.3%)	0.6% (2/329)	–	0.35 (0.08-1.13)

**Sex, *n* (%)**					
Men	1841 (61.4%)	1955 (61.1%)	3.4% (67/1955)	–	1.00
Women	1157 (38.6%)	1244 (38.9%)	3.8% (47/1244)	–	1.20 (0.79-1.80)

**Social grade, *n* (%)**					
C2DE	695 (23.2%)	817 (25.5%)	4.4% (36/817)	–	1.00
ABC1	2303 (76.8%)	2381 (74.5%)	3.3% (79/2381)	–	1.05 (0.66-1.74)

**Frequency of internet access, *n* (%)**					
Never	89 (3.0%)	79 (2.5%)	2.5% (2/79)	–	1.00
Rarely	151 (5.0%)	142 (4.4%)	4.2% (6/142)	–	1.09 (0.20-8.20)
Frequently	2758 (92.0%)	2977 (93.1%)	3.6% (106/2977)	–	1.52 (0.43-9.70)

**Motivation to reduce drinking, *n* (%)**					
Low	2391 (79.8%)	2549 (79.7%)	2.7% (70/2549)	–	1.00
High	607 (20.2%)	650 (20.3%)	6.8% (44/650)	–	2.49 (1.63-3.77)***
**AUDIT, mean (*SD*)**	10.4 (*5.0*)	10.5 (*5.7*)	–	–	1.07 (1.03-1.11)***

*Note.*^a^ Unweighted; ^b^ Weighted using the rim (marginal) technique to match the sample to the proportions of the English population profile on the dimensions of age, social grade, region, tenure, ethnicity and working status within sex; * *p* < 0.05; ** *p* < 0.01; *** *p* < 0.001.

In weighted analyses, 2.7 % (95 % CI = 2.2%–3.0%) of smokers (website: 2.5 %; app: 0.6 %) and 3.6 % (95 % CI = 2.9%–4.0%) of drinkers (website: 2.5 %; app: 1.2 %) reported that they had used a digital aid in a recent quit/reduction attempt. These proportions did not significantly differ (*p* = 0.06). In the sensitivity analysis, 2.4 % (95 % CI = 1.5%–4.0%) of recent ex-smokers reported that they had used a digital aid in a recent quit attempt (see Supplementary File 1).

In weighted univariable analyses, survey year was not significantly associated with the use of a digital aid in either a recent quit attempt in smokers (see Table 1) or a reduction attempt in drinkers (see Table 2). In the sensitivity analysis, the non-significant association between survey year and the use of a digital aid in a quit attempt remained in recent ex-smokers (see Supplementary File 1).

None of the smoking or sociodemographic variables of interest were significantly associated with the use of a digital aid in a recent quit attempt in smokers (see Table 1). In drinkers, being highly motivated to reduce drinking (OR_adj_ = 2.49, 95 % CI = 1.63–3.77, *p* < .001) and having a higher AUDIT score (OR_adj_ = 1.07, 95 % CI = 1.03–1.11, *p* < .001) were positively associated with the use of a digital aid in a recent reduction attempt (see Table 2).

## 4 Discussion

### 4.1 Principal findings

Adoption rates of digital aids for smoking cessation and alcohol reduction were low among smokers and drinkers who had made a past-year quit/reduction attempt (2.7 % of the 26.9 % who had made a quit attempt and 3.6 % of the 15.3 % who had made a reduction attempt, respectively) in a representative sample in England. The adoption rates did not significantly differ between groups, although adoption was nominally greater in drinkers. No significant year-on-year trends in adoption rates were detected between 2015 and 2018 in either smokers or drinkers. None of the sociodemographic or smoking characteristics of interest were significantly associated with the adoption of digital aids in smokers. Drinkers with high (compared with low) motivation to reduce alcohol consumption and higher AUDIT scores had greater odds of adoption of digital aids.

To estimate the public health impact of digital aids, researchers need to consider both effectiveness and reach within the target population. The observation that less than 5 % of smokers and drinkers report having used a digital aid for smoking cessation and alcohol reduction in a recent cessation/reduction attempt indicates that digital aids are not yet reaching a substantial proportion of the target population in England. One apparent explanation for the low adoption rates is a lack of public awareness. However, public-facing smoking cessation and alcohol reduction campaigns, such as the annual ‘Stoptober’ campaign (Brown et al., 2014), the annual ‘Dry January’ campaign (Ballard, 2016) and the year-round ‘One You’ campaign (Public Health England, 2016), have heavily promoted the use of smartphone apps for smoking cessation and alcohol reduction since 2016 and 2017, respectively. The effects of these campaigns on the public awareness of digital aids or crude adoption rates are unclear. The Smoking and Alcohol Toolkit Studies are limited by not currently including measures of awareness of available aids. Hence, future research is required to establish whether the low adoption rates are attributable to low awareness, or whether other factors are more influential.

We did not observe a significant difference in adoption rates between smokers and drinkers, although adoption was nominally greater in drinkers. Previous research has described the use of aids for smoking cessation and alcohol reduction in England and indicates that smokers are more likely than high-risk drinkers to use any form of cessation/reduction support, including pharmacotherapy and face-to-face behavioural support (Beard et al., 2016): data from England indicate that 60.3 % of smokers used a cessation aid in the past year, compared with 14.9 % of high-risk drinkers. The lack of differential adoption rates of digital aids between groups in the present study suggests that diffusion does not appear to be occurring unequally across smokers and drinkers.

The lack of an association between survey year and adoption rates in both smokers and drinkers may be interpreted to suggest that diffusion has stalled or is occurring too slowly between 2015 and 2018 to be detected. The ability to detect an association may also have been hindered by adoption being a rare event in this sample. Future research should continue monitoring adoption rates, with a view to examining trends over a longer period of time.

A previous comparison of the characteristics of app users with those of the general population of smokers in England indicated that app users were more likely to be younger, female, have a non-manual occupation, and have higher daily cigarette consumption (Ubhi et al., 2015). The lack of associations between smoking and sociodemographic characteristics and the use of a digital aid among smokers in the present study leave open the question of whether the process of diffusion has occurred unequally across different social groups. However, non-significant trends were observed whereby older adults had reduced odds and those from social grades ABC1 had increased odds of adoption, thus suggesting that other datasets should be explored for these trends.

The finding that alcohol consumption, as measured by the AUDIT, was positively associated with the adoption of digital aids in high-risk drinkers echoes previous research. For example, users of the Drinks Meter app, compared with drinkers in the general population in England, had a higher mean AUDIT score (Garnett et al., 2017). Users who were willing to be randomised in a factorial trial of the Drink Less app had a mean age of 39.2 years and a mean AUDIT score of 19.1 (Crane et al., 2018), which is indicative of harmful drinking. Although previous studies have not assessed drinkers’ level of motivation, our findings suggest that the diffusion process has occurred at a faster rate in those who are highly motivated to reduce their drinking. Hence, strategies to accelerate the diffusion process may benefit from specifically targeting those with lower motivation to reduce their alcohol consumption. The nominally greater uptake of digital aids in drinkers may be partly related to 74.5 % of drinkers who had made an attempt to reduce their consumption being from social grades ABC1, as compared with 40.7 % of smokers who had attempted to quit. Results from a previous comparison of the characteristics of users of the Drinks Meter app with the general population of drinkers in England indicated that app users had reduced odds of being from a lower social grade (Garnett et al., 2017). However, in the current study, smokers and drinkers from social grades ABC1 compared with C2DE had numerically but not significantly increased odds of adoption.

### 4.2 Strengths and limitations

To the authors’ knowledge, this was the first study to examine trends in and factors associated with the adoption of digital aids for smoking cessation and alcohol reduction in a large, representative sample of smokers and drinkers in England. However, this study was limited by only asking about the use of digital aids in serious attempts to quit smoking/reduce alcohol consumption. Asking about ever-use of digital aids may provide a more accurate estimate of adoption rates in future research. Evidence suggests that a large proportion of unsuccessful smoking cessation attempts fail to be reported, particularly if they only last a short time or occurred a longer time ago (i.e. more than 3 months ago) (Berg et al., 2010). As approximately 90 % of health and fitness app users disengage with their selected app one week after having downloaded it (Appboy, 2016; Consumer Health Information Corporation, 2015), it is plausible that a substantial proportion of smokers and drinkers forget quit/reduction attempts involving a digital aid. Future research is needed to establish whether differential reporting of quit attempts may be occurring as a function of the use of various cessation aids (Berg et al., 2010).

To allow for a direct comparison of smokers and drinkers, both groups were asked about having made a serious quit/reduction attempt. However, drinkers may be more inclined to think about restricting their consumption, as opposed to making a serious attempt to reduce drinking, which may be perceived as qualitatively different. Indeed, a greater proportion of respondents in the Alcohol Toolkit Study report that they are currently trying to restrict their consumption (i.e. 25–30%; http://www.alcoholinengland.info/) than those who report that they have made a serious attempt to reduce their drinking (i.e. 15 %). It is therefore plausible that the true adoption rate of digital aids amongst drinkers is higher than the proportion estimated in the current study, which should be treated as a conservative estimate. Another limitation is that we were unable to take account of multiple quit/reduction attempts. Although smokers in the Smoking Toolkit Study are asked about aids used in up to three quit attempts, drinkers in the Alcohol Toolkit Study are only asked about aids used in their most recent attempt. To allow for a direct comparison, we only included data from the most recent attempt in both smokers and drinkers. As such, our findings likely do not reflect first use of a digital aid to support a quit/reduction attempt. It is also possible that smokers and drinkers may have used digital aids not captured by the survey (e.g. a computer programme).

### 4.3 Implications

Despite these limitations, this study has important implications for digital health research, practice and policy. Although several theory- and evidence-informed digital aids for smoking cessation and alcohol reduction (e.g. websites, smartphone apps) have been found to be effective in controlled trials (Kaner et al., 2017; Taylor et al., 2017), researchers and policy-makers may consider devising public health campaigns to increase the awareness of evidence-based digital aids. Recent efforts have been made to develop regulatory frameworks for digital aids in the UK with related resources (e.g. the National Health Service’s Apps Library; https://www.nhs.uk/apps-library/) specifically designed to help practitioners and patients navigate the host of available apps. Increased awareness of and the confidence to use such resources amongst practitioners and members of the public may also help to speed up the diffusion process.

In addition, research assessing for whom, in what contexts and why particular digital aids are likely to be effective is still in its infancy. For example, ‘guided’ or ‘blended’ interventions (i.e. those offering a combination of digital and face-to-face support) have been found to be superior to stand-alone digital aids for depression and anxiety (Kleiboer et al., 2015; Richards and Richardson, 2012) and alcohol (Riper et al., 2018). In a recent international, cross-sectional survey of regular drinkers, higher-risk drinkers expressed a preference for face-to-face, specialist support (Davies et al., 2019). Researchers, practitioners and policy-makers should hence consider devising and evaluating different implementation strategies, including the promotion of digital aids alongside face-to-face support, to ensure that effective digital aids are integrated into routine health services and that additional, specialist support is available for higher-risk drinkers if needed (Hermes et al., 2019).

## 5 Conclusion

The adoption of digital aids was low in both smokers and drinkers, did not differ between groups and was not positively associated with survey year. No associations between the sociodemographic and smoking characteristics of interest and the adoption of digital aids in smokers were detected, while drinkers who were highly motivated to reduce their consumption and those with higher AUDIT scores had greater odds of adoption. Researchers and policy-makers should consider devising public health campaigns to increase awareness of digital aids and implementation strategies to ensure effective digital aids are integrated into routine health services.

## Role of funding source

Cancer Research UK is the main contributor (C1417/A22962). The UK Department of Health, Pfizer, GlaxoSmithKline and Johnson and Johnson have also all contributed funding to the data collection for the Smoking Toolkit Study. The National Institute for Health Research’s School for Public Health Research has contributed funding to the Alcohol Toolkit Study. The funders had no final role in the study design; in the collection, analysis and interpretation of data; in the writing of the report; or in the decision to submit the paper for publication. All researchers listed as authors are independent from the funders and all final decisions about the research were taken by the investigators and were unrestricted.

## Contributors

All authors contributed to the design of the study. OP conducted the statistical analysis and wrote the first draft of the manuscript. All authors have contributed to and have approved the final manuscript.

SEJ and CG have no conflicts of interest to declare. OP, JB and RW are unpaid members of the scientific steering group of the Smoke Free app. RW undertakes research and consultancy for and receives travel funds and hospitality from manufacturers of smoking cessation medications (Pfizer, GlaxoSmithKline and Johnson and Johnson), who have financial interests in digital aids for smoking cessation, but not alcohol reduction. JB has received unrestricted funding for smoking cessation research from Pfizer.

## Declaration of Competing Interest

SEJ and CG have no conflicts of interest to declare. OP, JB and RW are unpaid members of the scientific steering group of the Smoke Free app. RW undertakes research and consultancy for and receives travel funds and hospitality from manufacturers of smoking cessation medications (Pfizer, GlaxoSmithKline and Johnson and Johnson), who have financial interests in digital aids for smoking cessation, but not alcohol reduction. JB has received unrestricted funding for smoking cessation research from Pfizer.
