# Supplementary material for: Trends in and factors associated with the adoption of digital aids for smoking cessation and alcohol reduction: A population survey in England
Source: Drug Alcohol Depend. 2019 Dec 1;205:107653. doi: 10.1016/j.drugalcdep.2019.107653 (PMC6905148; doi:10.1016/j.drugalcdep.2019.107653)
Supplement: Supplementary file 1 [file mmc1.docx]

**Supplementary Material for the Article:**

Trends in and factors associated with the adoption of digital aids for smoking cessation and alcohol reduction: A population survey in England

**This material supplements, but does not replace, the peer-reviewed paper in**

***Drug and Alcohol Dependence*.**

Olga Perski^a^, Sarah E. Jackson^a^, Claire Garnett^a^,
Robert West^a^, Jamie Brown^a^

^a^Department of Behavioral Science and Health, University College London, 1-19 Torrington Place, London WC1E 6BT, UK

**Correspondence:**

Olga Perski
Department of Behavioral Science and Health, University College London
1-19 Torrington Place, London WC1E 6BT, UK

Phone: +44(0)20 7679 1258
olga.perski@ucl.ac.uk

*Supplementary File 1.* Recent ex-smokers’ characteristics in the unweighted and weighted datasets and Odds Ratios (ORs) from the weighted univariable analysis.

|  | **Recent ex-smokers^a^**  **(*N* = 836)** | **Recent ex-smokers^b^**  **(*N* = 899)** | **% Used a digital aid in recent attempt^b^ (*n/N*)** | **OR (95% CI)** |
| --- | --- | --- | --- | --- |
| **Used a digital aid in recent attempt, *n* (%)** |  |  |  |  |
| No | 817 (97.7%) | 877 (97.6%) | - | - |
| Yes | 19 (2.3%) | 22 (2.4%) | - | - |
| **Survey year, *n* (%)** |  |  |  |  |
| 2015 | 227 (27.2%) | 243 (27.0%) | 3.7% (9/243) | 1.00 |
| 2016 | 220 (26.3%) | 241 (26.8%) | 2.1% (5/241) | 0.48 (0.14-1.66) |
| 2017 | 228 (27.3%) | 244 (27.1%) | 2.5% (6/244) | 0.64 (0.20-2.07) |
| 2018 | 161 (19.3%) | 170 (18.9%) | 1.2% (2/170) | 0.29 (0.05-1.59) |
| **Age, *n* (%)** |  |  |  |  |
| 16-24 | 158 (18.9%) | 156 (17.4%) | 2.6% (4/156) | - |
| 25-34 | 207 (24.8%) | 251 (27.9%) | 3.6% (9/251) | - |
| 35-44 | 142 (17.0%) | 173 (19.2%) | 2.9% (5/173) | - |
| 45-54 | 131 (15.7%) | 153 (17.0%) | 3.3% (5/153) | - |
| 55-64 | 112 (13.4%) | 96 (10.7%) | 0% (0/96) | - |
| 65+ | 86 (10.3%) | 70 (7.8%) | 0% (0/70) | - |
| **Sex, *n* (%)** |  |  |  |  |
| Men | 441 (52.8%) | 483 (53.7%) | 2.5% (12/483) | - |
| Women | 395 (47.2%) | 415 (46.2%) | 2.4% (10/415) | - |
| **Social grade, *n* (%)** |  |  |  |  |
| C2DE | 393 (47.0%) | 449 (49.9%) | 2.4% (11/449) | - |
| ABC1 | 443 (53.0%) | 450 (50.1%) | 2.4% (11/450) | - |
| **Frequency of internet access, *n* (%)** |  |  |  |  |
| Never | 45 (5.4%) | 40 (4.4%) | 2.5% (1/40) | - |
| Rarely | 56 (6.7%) | 61 (6.8%) | 1.6% (1/61) | - |
| Frequently | 735 (87.1%) | 797 (88.7%) | 2.5% (20/797) | - |

*Note.* ^a^ Unweighted; ^b^ Weighted; * *p* < .05; ** *p* < .01; *** *p* < .001.
